# A dataset for two-echelon electric vehicle routing problems

**DOI:** 10.1016/j.dib.2025.111470

**Published:** 2025-03-15

**Authors:** Mehmet Anıl Akbay, Christian Blum

**Affiliations:** Artificial Intelligence Research Institute (IIIA-CSIC), Campus of the UAB, Bellaterra, 08193, Spain

**Keywords:** Electric vehicle routing problem, Two-echelon, Time windows, Simultaneous pickup and deliveries, Partial delivery

## Abstract

This paper introduces a dataset generated for research on Two-Echelon Electric Vehicle Routing Problems (2E-EVRPs) with additional constraints, including time windows, simultaneous pickup and delivery (SPD), and partial deliveries. The dataset is derived from established benchmark instances from the VRP and EVRP literature and further extended using methodologies from the literature. It features diverse scenarios designed to challenge and validate solution approaches proposed for two-echelon routing algorithms under various constraints.

The dataset comprises a variety of instances, ranging from small (5-15 customers) to large (100 customers), and incorporates different geographical configurations, including clustered, random, and random-clustered distributions. Key modifications include integrating satellite locations, enhanced vehicle configurations concerning electric vehicle (EV) constraints, and adjusting time windows to accommodate the two-echelon structure. The dataset also supports multiple delivery scenarios, allowing for single delivery, simultaneous pickups and deliveries (SPD), and partial deliveries, enabling researchers to test the performance of their algorithms across a range of realistic constraints.

Specifications Table

**Table:** 

Subject	Operations Research, Sustainable Logistics
Specific subject area	Two-echelon Electric Vehicle Routing Problems belong to the family of Vehicle Routing Problems (VRPs). They combine electric vehicle routing with a multi echelon distribution strategy as a sustainable logistics practice.
Type of data	Text file with tables (.txt)
Data collection	Nodes include coordinates for the central warehouse, satellites, charging stations, and customers. Each node is defined with features such as demand and time windows, adapted for various delivery and pickup scenarios, including single delivery, simultaneous pickup and delivery (SPD), and partial deliveries. Vehicle information includes loading capacities for large vehicles in the first echelon and smaller electric vehicles in the second. Electric vehicles have battery capacities, inverse refuelling rates, costs per travelled distance, and energy consumption per travelled distance.
Data source location	Benchmark instances were generated based on the Electric Vehicle Routing Problem (EVRP) instances originally proposed in Schneider et al. (2014), with modifications to adapt to a two-echelon distribution structure and considering various delivery and pickup scenarios.
Data accessibility	Repository name: Zenodo Data identification number: https://doi.org/10.5281/zenodo.14844216 Direct URL to data: https://zenodo.org/records/14844216
Related research article	The presented benchmark instances were introduced in the following research paper: Akbay et al. [1] “Variable Neighborhood Search for the Two-Echelon Electric Vehicle Routing Problem with Time Windows.” Applied Sciences. DOI: https://doi.org/10.3390/app12031014

## 1 Value of the Data

• **Unique Constraints:** This dataset includes multiple constraints, including the two-echelon structure, time windows, simultaneous pickup and delivery, and partial deliveries, which are not jointly covered in existing datasets.
• **Broader Application:** The dataset enables comparative analyses on multi-echelon routing models, supporting advancements in algorithms for green logistics.
• **Reusable for Scenario Variations:** Researchers can easily modify customer demands, vehicle capacities, and satellite depot positions to test algorithm performance across varying logistics scenarios.
• **Facilitates Benchmarking:** This dataset contributes to the benchmark sets in the vehicle routing literature, allowing for standardized evaluation of solution methods targeting sustainable logistics.

## 2 Background

The Vehicle Routing Problem (VRP), originally formulated by Dantzig and Ramster [4], is at the core of logistics problems in which a fleet of vehicles must optimally serve a set of customers from one or multiple depots. The VRP is a multi-faceted problem with objectives such as minimizing travel distance, time, or operational costs, and has evolved into numerous variants to address real-world complexities.

**Fig. 1:**
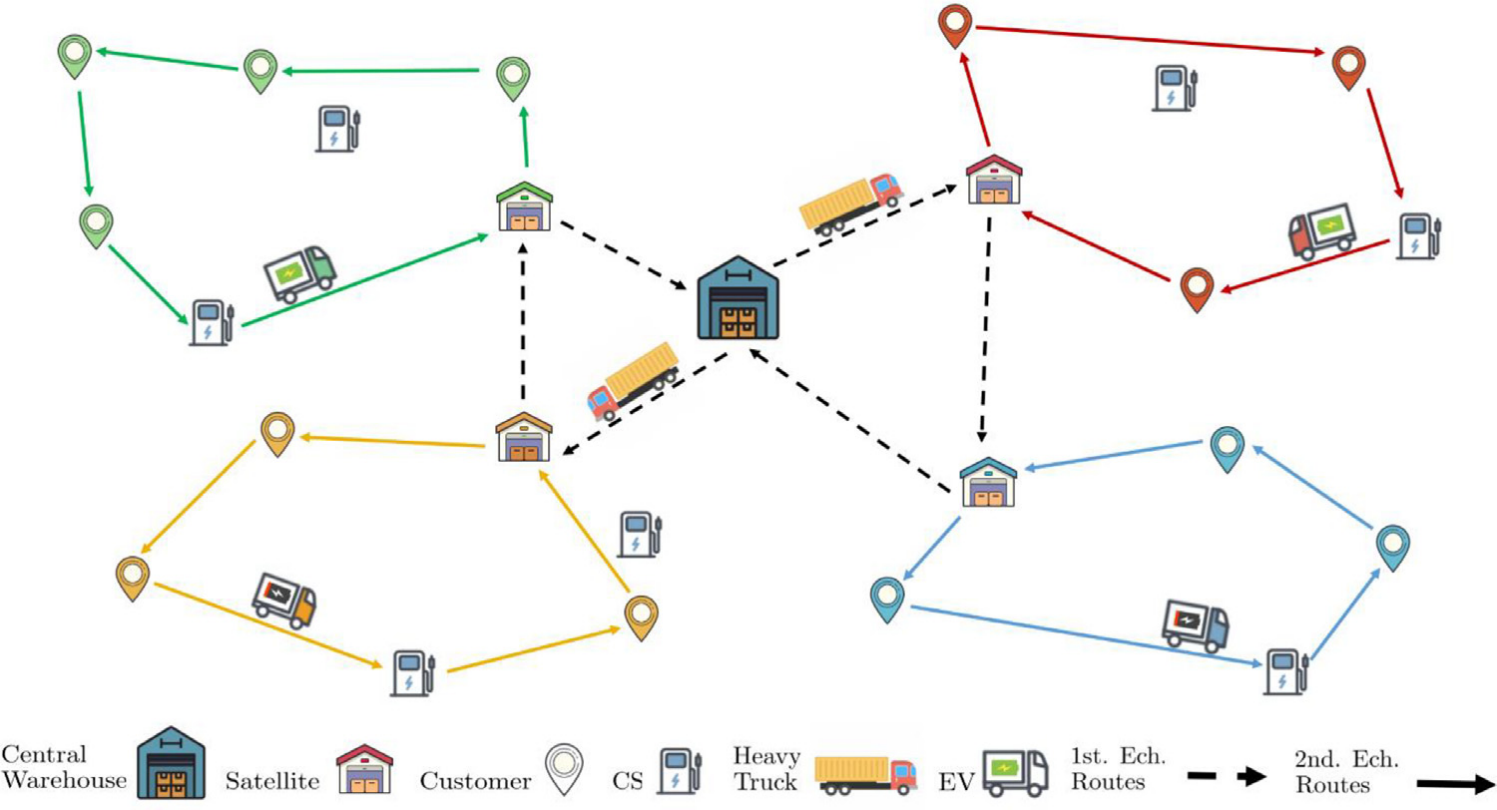
Illustration of a 2E-EVRP problem instance. Icons by Icons8 (icons8.com).

One significant variant, the Electric Vehicle Routing Problem (EVRP), introduces the use of electric vehicles (EVs) in distribution networks, emphasizing sustainable logistics through reduced emissions and energy consumption. Compared to conventional VRPs, the EVRP must cope with additional challenges, including limited battery range and recharging requirements at charging stations [3].

In response to the unique needs of urban logistics, the Two-Echelon Vehicle Routing Problem (2E-VRP) divides delivery operations into two levels. Goods are transported from a central warehouse (CW) to intermediate transshipment facilities, in other words, satellites in the first echelon, and then smaller vehicles deliver from satellites to final customer locations within city centers in the second echelon [6]. This structure accommodates complex urban constraints while maintaining an efficient distribution of goods.

Combining the concepts of 2E-VRP and EVRP, the Two-Echelon Electric Vehicle Routing Problem (2E-EVRP) leverages the sustainability of EVs within a two-echelon framework [2]. Large trucks transport goods from the central warehouse to satellites in the first echelon, while the second echelon uses EVs to navigate urban areas, meeting customer demands under constraints such as time windows, vehicle capacities, and charging station accessibility (Fig. 1). This multi-level approach supports the sustainable, efficient operation of urban logistics networks.

## 3 Data Description

This section presents the final configurations of the instances by providing an example structure of the instance text files, figures depicting the instances visually, and explanations of the indexing system used for naming the instance files.

**1. Instance Groups:**

The generated dataset consists of three main groups, categorized by the number of customers and the configuration of satellite depots:

a. **Small-Scale Instances:** 5 or 10 customers, 1 satellite depot at (50,75).
b. **Medium-Scale Instances:** 15 customers with 2 satellite depots at grid intersections (50,25), (50,75), and 50 customers with 4 satellites at grid intersections (25,25), (75,25), (25,75), and (75,75).
c. **Large-Scale Instances:** 100 customers with 8 satellite depots arranged at grid intersections (25,25), (25,50), (25,75), (50,25), (50,75), (75,25), (75,50), and (75,75).

**2. Instance Naming Convention:**

**Fig. 2:**
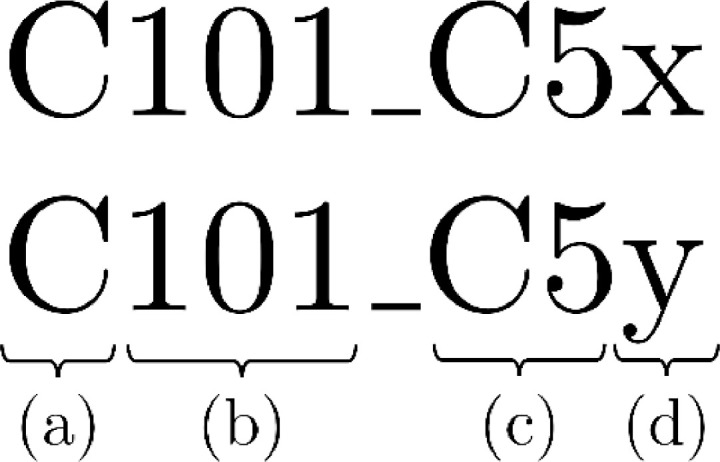
Naming convention used for the problem instance files. Each file name consists of four main sections: (a) Prefix indicating the spatial distribution of customer locations (C: Clustered, R: Random, RC: Random-Clustered), (b) Original dataset code (e.g., 101), (c) The number of customers in the dataset (e.g., C5), (d) Two different versions created for the pickup and delivery models (labeled as x and y).

The naming convention for each instance file provides the complete instance information in text format, as illustrated in Fig. 2. The name of each instance file begins with a prefix indicating the spatial distribution of the customer locations. Three main prefixes are used: ``C'' for a clustered distribution, simulating densely populated or urban environments; ``R'' for a random distribution, representing more spread-out or rural areas; and ``RC'' for a random-clustered distribution, which combines both clustered and random placements to reflect varied real-world logistics scenarios.

Following the prefix, an instance number (e.g., 101, 103) is included to distinguish between different instances within the same spatial distribution type. For example, instances labeled 101 and 103 have different configurations of customer locations and other parameters despite sharing the same distribution pattern.

The next component in the naming structure specifies the number of customers in the instance, denoted as ``C5'' for instances with 5 customers, or ``C15′' for instances with 15 customers.

Finally, the scenario version is appended as either ``x'' or ``y'' to indicate variations in demand configuration. The ``x'' version represents a straightforward setup where delivery and pickup demands are applied directly for each customer. In contrast, the ``y'' version introduces variability by adjusting the ratios of pickup and delivery demands, creating a diverse set of scenarios that allow for more comprehensive testing and analysis across different logistical conditions.

3. **Instance File Structure:**

Each file is divided into two main sections: the node information and the vehicle configuration. The first section starts with a header line and contains the details for each node in the problem instance. Each node is specified by a unique identifier (StringID), its type (e.g., ``d: central warehouse'', ``s: satellite'', ``f: charging station'', or ``c: customer''), and its Cartesian coordinates. Demand-related information is included, such as single demand for basic delivery problems, separate or simultaneous delivery and pickup demands for scenarios involving reverse logistics, and a division rate column to handle partial delivery scenarios where customer demand can be split between vehicles. Additionally, the time windows for each node are defined by the ``ReadyTime” and ``DueDate'' columns, and the ``ServiceTime'' indicates the duration required to serve the node.

The second section of the file specifies the vehicle configuration parameters. It includes the loading capacities for both large vehicles used in the first echelon and smaller electric vehicles in the second echelon. Battery capacity, fuel consumption rates, inverse refueling rates, and average velocity are also provided, allowing for detailed electric vehicle operations and constraints modeling.

**Fig. 3:**
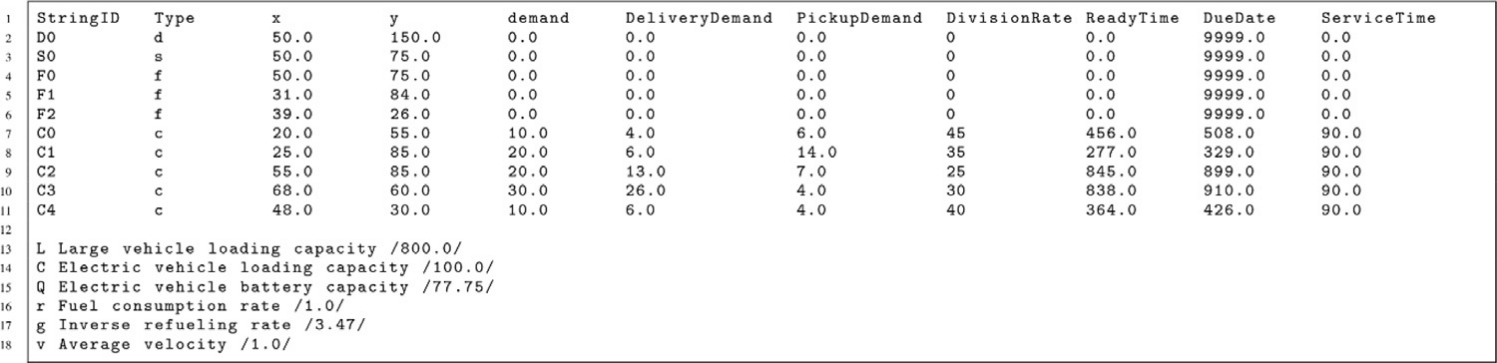
Example instance file.

Fig. 3 provides an example of the file format, illustrating the node information structure and the vehicle configuration parameters.

## 4 Experimental Design, Materials and Methods

### 4.1 Original dataset

This dataset aims to support research on the 2E-EVRP by providing a structured set of instances considering various logistical constraints, including time windows and diverse demand scenarios such as single delivery, simultaneous pickup and delivery (SPD), and partial delivery scenarios. The instances of the dataset were generated based on well-established benchmark instances from the EVRP literature, allowing for robust comparative analysis across different routing methods and configurations.

The original EVRP-TW dataset, derived by Schneider et al. [9] from the classical VRP-TW instances by Solomon [10], includes a total of 92 instances: 36 small-sized instances with 5, 10, or 15 customers, and 56 large-sized instances containing 100 customers and 21 charging stations. Each instance is organized into three main groups, reflecting different spatial distributions of customer locations:

• **Clustered (prefix ``C''):** Customers are located in groups of clusters, simulating a densely populated or urban environment.
• **Random (prefix ``R''):** Customer locations are randomly scattered, representing more spread-out, respectively rural, scenarios.
• **Random-Clustered (prefix ``RC''):** With a mix of clustered and randomly distributed customer locations, this hybrid setup reflects diverse real-world logistics scenarios with varied customer density.

Each group also contains sub-categories—called Type 1 and Type 2—differentiating instances by parameters such as planning horizons, time windows, vehicle load, and battery capacities, which affect fleet configuration and routing structures.

Schneider's modifications primarily involved integrating charging stations and adjusting battery capacities to ensure instance feasibility. Specifically, one charging station was positioned at the depot, with the remaining stations distributed randomly, yet in such a way that every customer could be reached using at most two charging stations. The battery capacity was determined as the maximum of (1) the need to travel 60% of the average route length of the best-known VRP solution and (2) twice the battery capacity required to traverse the longest arc from a customer to a charging station. These changes also caused the creation of new time windows, as the original ones from Solomon [10] became infeasible due to added constraints related to charging times.

**Fig. 4:**
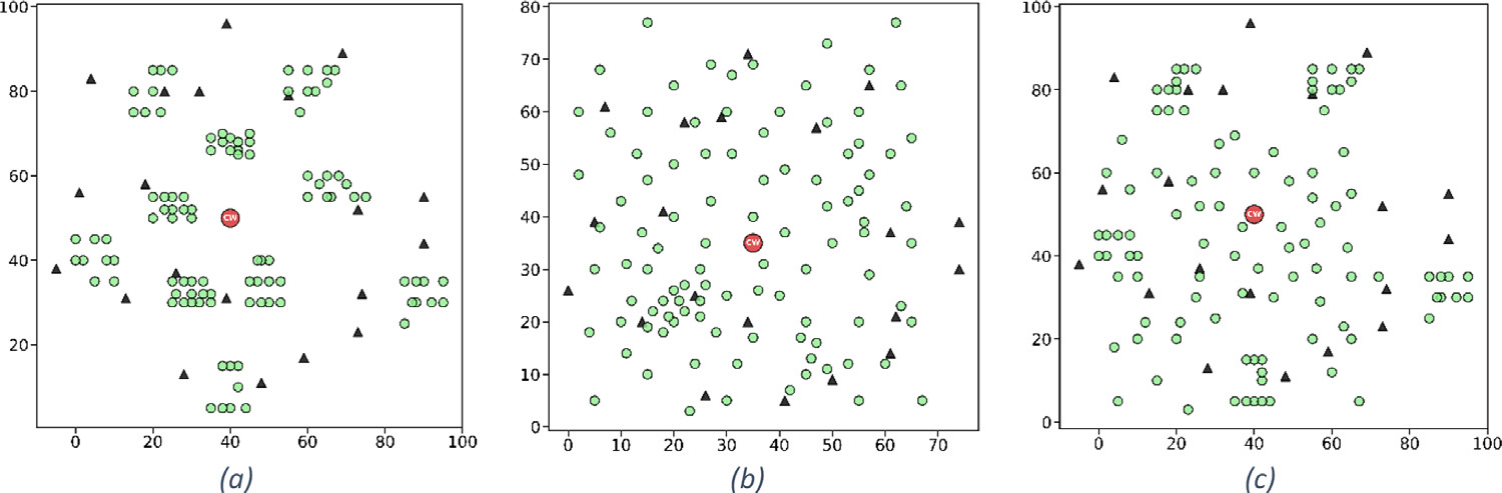
Illustration of three example instances from the original dataset [9] for large-sized instances, highlighting diverse spatial distributions. Each instance depicts customer and facility layouts for different characteristics: (a) clustered, (b) random, and (c) random-clustered arrangements. In all subfigures, green circles represent customers, black triangles indicate charging stations, and the red circle marks the central warehouse.

Fig. 4 depicts three examples from the original dataset for large-sized instances with clustered, random, and random-clustered customers.

### 4.2 Generated dataset

As mentioned before, the problem instances of our dataset consider a two-echelon distribution network with additional operational constraints. Therefore, the existing problem instances required significant restructuring. Following the methodology of Grangier et al. [5], each problem instance was extended to integrate satellite depots alongside the central warehouse. Moreover, additional changes were made to reflect real-world complexities such as varying vehicle configurations, demand types, and geographical settings. Details of this process, including the geographical layout, demand reconfiguration, time window adjustment, vehicle capacity recalibration, and changes to the charging infrastructure, are presented in the following sections.

#### 4.2.1 Geographical configuration

**Fig. 5:**
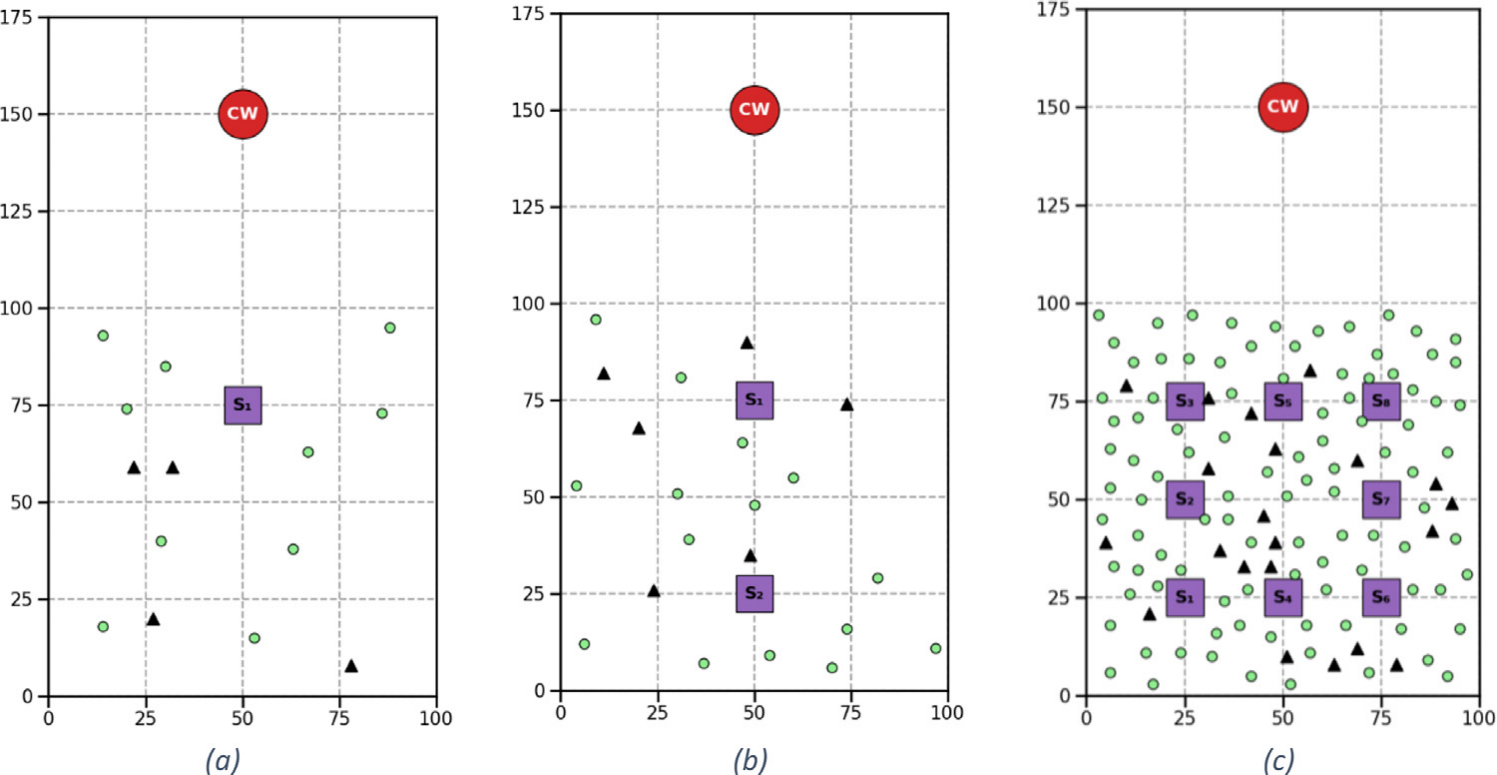
Illustration of the grid-based layout for the central warehouse, satellites, customers, and charging stations across different cases. The central warehouse is located at coordinates (50, 150) in all problem instances. Green circles represent customers, and black triangles indicate charging stations. Customer and charging station positions are randomly generated for illustration purposes. (a) Small instance with 1 satellite located at (50, 75) and 10 customers. (b) Medium-size instance with 2 satellites positioned at (50, 75) and (50, 25), along with 15 customers. (c) Large instance featuring 8 satellites at locations (25, 25), (25, 50), (25, 75), (50, 25), (50, 75), (75, 25), (75, 50), and (75, 75), with 100 customers.

Each problem instance defines the locations of the central warehouse and satellites on a two-dimensional grid, with coordinates proportionally scaled according to map dimensions. The placement follows a systematic approach, ensuring that satellite depots are uniformly distributed at the grid intersections, providing balanced spatial coverage for customer locations across different instance scales (see Fig. 5).

For large-scale datasets involving 100 customers, satellite depots are positioned at the following eight evenly spaced grid intersections: (25,25), (25,50), (25,75), (50,25), (50,75), (75,25), (75,50), and (75,75). This arrangement ensures comprehensive spatial coverage, balancing logistical demands across urban and peripheral areas and enabling efficient routing between depots and customer locations.

In small datasets, with 5 or 10 customers, a single satellite depot is sufficient to meet demand. This satellite depot is positioned centrally within the grid at (50,75), ensuring accessibility and minimizing travel distance for smaller-scale deliveries. For datasets with 15 customers, configurations include both single and dual satellite setups, providing flexibility in routing scenarios. In instances with two satellite depots, these are placed at (50,75) and (50,25), offering a wider spatial distribution to handle increased customer demands effectively.

In addition to the small and large-sized instances, we have generated middle-sized instances containing 50 customers. This is done by systematically removing some of the customers from each large-sized instance. These instances have 4 satellites placed at (25,25), (75,25), (25,75), and (75,75).

#### 4.2.2 Time window adjustments

With the two-echelon structure, adjustments to the original time windows were essential to maintain feasibility given the extended depot configuration. The central warehouse's relocation to a more peripheral position on the grid introduces potential discrepancies between the original time windows and the increased travel distances required in this setup. This shift in depot location can result in time window violations for some customers if not appropriately adjusted.

To address this, a time shift (Δ) was introduced to each customer's original time window $\left[a_{i},b_{i}\right]$. This shift accounts for the extra travel time required between the new central warehouse (CW) and the original depot position (D), ensuring that time windows remain aligned with the two-echelon structure. Mathematically, this adjustment can be represented as:

$$\left[a_{i}+\Delta ,\;b_{i}+\Delta \right]$$

Where Δ=⌈t(CW,D)⌉ represents the added travel time between the newly positioned central warehouse and the depot location from the original problem instance, calculated based on Euclidean distance between the respective locations. This approach extends time windows as necessary to accommodate the greater distances the reconfiguration introduces. By incorporating these modifications, the dataset retains the feasibility of customer service schedules across the two-echelon structure.

#### 4.2.3 Vehicle configuration

Vehicle capacities were calibrated in each echelon based on the original configurations found in the Schneider dataset, ensuring consistency with established capacity-constrained and time-constrained characteristics. Following the classification used by Savelsbergh [8], the instances labeled with a ``1'' (R1, C1, RC1) represent cases with short scheduling horizons, while instances labeled with a ``2'' (R2, C2, RC2) feature longer scheduling horizons. This distinction determines the loading capacities of the vehicles serving in both echelons.

• **Short Scheduling Horizons (Type 1 Instances):** In instances with shorter time frames, where time constraints are tighter, we applied a vehicle capacity ratio of 4/0.5 for first-level (long-haul trucks) to second-level EVs. This setup allows first-level vehicles to transport larger loads from the central depot to the satellite depots, while second-level EVs are configured with smaller capacities for efficient distribution to final destinations within city limits.
• **Long Scheduling Horizons (Type 2 Instances):** For instances with extended planning horizons, characterized by more relaxed time constraints and a focus on load capacity, a capacity ratio of 2/0.25 was used. This ratio provides ample carrying capacity for both levels while focusing on maximizing vehicle utilization within the two-echelon structure, allowing EVs to handle more substantial portions of demand without exceeding battery or load limitations.

These ratios align the dataset with the dual challenges of maintaining efficient load distribution in time-sensitive scenarios and optimizing vehicle use in capacity-sensitive instances. They support realistic testing of algorithms under different operational conditions, ensuring that both time and load constraints are appropriately addressed across two distinct routing levels.

#### 4.2.4 Demand configuration

In real-world logistics distribution, different scenarios necessitate diverse demand configurations. We have made specific adjustments to the original problem instances to accommodate these variations.

##### 4.2.4.1 Demand modifications for pickup and delivery problems

In logistics, while some cases involve only delivery operations, other scenarios require vehicles to collect goods from certain demand points. For instance, in e-commerce logistics, handling returned products is a form of reverse logistics. We introduced two distinct demand components in our dataset to capture these complexities: *delivery demand* and *pickup demand*. These components can be utilized in problem variants that involve both delivery and pickup operations, such as backhauls, linehauls, pickup and delivery problems, or simultaneous pickup and delivery scenarios.

Since Schneider's original instances only provided a single demand type per customer, we separated the combined demand into delivery and pickup components. To achieve this, we applied the method described by Salhi and Nagy [7], using each customer $c_{i}$’s Cartesian coordinates $\left(x_{i},y_{i}\right)$ to calculate a ratio ρ, defined as:

$$\rho_{i}=min\left\{\frac{x_{i}}{y_{i}},\frac{y_{i}}{x_{i}}\;\right\}$$

The ratio $\rho_{i}$ was then used to derive the delivery demand $q_{i}\;$by multiplying the original demand $\delta_{i}$ by $\rho_{i}$:

$$q_{i}=\;\rho_{i}\;\times \;\delta_{i}$$

The pickup demand $p_{i}\;$for each customer was subsequently calculated by subtracting the delivery demand from the original demand:

$$p_{i}=\delta_{i}-\;q_{i}$$

This adaptation ensures that each customer's original demand is split realistically into separate delivery and pickup needs, enabling comprehensive modeling of the SPD constraints within the dataset.

To further enrich the dataset, we created two versions for each SPD-modified instance:

• **Version 'X' of the dataset:** This version includes the straightforward application of delivery and pickup demands for each customer.
• **Version 'Y' of the dataset:** This version alternates customer demands by adjusting pickup and delivery ratios, creating variation across datasets and allowing for a broader range of testing scenarios.

##### 4.2.4.2 Demand modifications for partial delivery scenarios

In addition to the delivery and pickup configurations, partial deliveries were incorporated to allow for greater flexibility in distribution. This modification allows for customer demands to be split and served by multiple vehicles to optimize delivery efficiency. In the original problem variant, each customer is served by a single vehicle; however, to allow for partial deliveries, an additional parameter, named *division rate*, was added to each instance.

The division rate for each customer was determined randomly, with values ranging between 20% and 50%. This rate specifies the proportion of the original demand that can be optionally handled by one vehicle, with the remaining demand assigned to another vehicle if needed. Formally, for a customer $c_{i}$ with an original demand $q_{i}$ and a division rate n%, the demand can be split such that:

$$\text{Partial}\;\text{Demand}=\;q_{i}\;\times \;\frac{n}{100}\;and\;\text{Remaining}\;\text{Demand}=\;q_{i}-\;Partial\;Demand$$

This setup allows for enhanced routing flexibility by enabling partial deliveries, making it feasible for each customer's demand to be served across multiple routes. Importantly, the dataset's constraints remain consistent with the original problem formulation, as the split demand approach is managed through the *division rate* parameter, which specifies permissible demand allocation across vehicles.

Through these adjustments, the dataset accommodates scenarios with pickup and deliveries and partial deliveries, supporting rigorous testing under complex, realistic conditions found in urban logistics.

#### 4.2.5 Charging station configuration

Remember that, in the original dataset by Schneider et al. [9], the central warehouse was one of the charging stations. Correspondingly, all the satellite depots are charging stations in our extended instances. Moreover, the central warehouse ceases to be a charging station as it is not frequented by EVs, only by long-haul trucks.

#### 4.2.6 Expanding the instances

To enhance the comprehensiveness of the dataset and support robust evaluations of solution methods, the number of problem instances in each category has been increased. The original dataset contained a limited number of instances in certain subsets. To address this limitation, we have expanded the dataset to include 50 problem instances for each group of customers (5, 10, 15, 50, and 100 customers) across all categories: C1, C2, R1, R2, RC1, and RC2. Table 1 illustrates the initial and current distribution of instances across different categories.

**Table 1:** Number of problem instances before and after dataset expansion.

Type	Num. of Customers	C1	C2	R1	R2	RC1	RC2	Total
Type x	5	2/50	2/50	2/50	2/50	2/50	2/50	12/300
	10	2/50	2/50	2/50	2/50	2/50	2/50	12/300
	15	2/50	2/50	2/50	2/50	2/50	2/50	12/300
	50	−/50	−/50	−/50	−/50	−/50	−/50	−/300
	100	9/50	8/50	12/50	11/50	8/50	8/50	56/300
	**Total**	**15/250**	**14/250**	**18/250**	**17/250**	**14/250**	**14/250**	**92/1500**
Type y	5	−/50	−/50	−/50	−/50	−/50	−/50	−/300
	10	−/50	−/50	−/50	−/50	−/50	−/50	−/300
	15	−/50	−/50	−/50	−/50	−/50	−/50	−/300
	50	−/50	−/50	−/50	−/50	−/50	−/50	−/300
	100	−/50	−/50	−/50	−/50	−/50	−/50	−/300
	**Total**	**−/250**	**−/250**	**−/250**	**−/250**	**−/250**	**−/250**	**−/1500**

After the expansion, each sub-category (C1, C2, R1, R2, RC1, RC2) contains 50 instances for each group of customers. This results in a total of 300 instances covering all customer group sizes.

##### 4.2.6.3 Methodology for instance generation

Generation of Large-Scale Instances (100 Customers): To expand the dataset, we first generated new instances with 100 customers, ensuring that every subgroup (C1, C2, R1, R2, RC1, RC2) contains 50 instances. These instances were created while preserving the defining characteristics of each original subgroup—clustered, randomly distributed, and random-clustered. Customer information, such as coordinates, demands, division rates, and time windows, was generated using random distributions that align with the statistical properties of the original datasets. This ensures the coherence and compatibility of the new instances with the existing dataset, making them suitable for various analytical methods, including machine learning.

Derivation of Smaller Instances: After generating the instances with 100 customers, we derived smaller-sized instances by systematically removing a specific number of nodes from the original datasets. Following methodologies from established EVRP and VRP datasets, we randomly removed nodes to create instances with 5, 10, 15, and 50 customers.

These enhancements result in a balanced and extensive dataset, facilitating objective evaluations of solution methods and supporting in-depth analytical research on dataset properties and solution characteristics.

## Limitations

Not applicable.

## Ethics Statement

Authors have read and follow the ethical requirements for publication in Data in Brief and confirming that the current work does not involve human subjects, animal experiments, or any data collected from social media platforms.

## CRediT Author Statement

**Mehmet Anıl Akbay:** Conceptualization of this study, Methodology, Software, Data curation, Writing – Original draft preparation. **Christian Blum:** Conceptualization of this study, Writing - Polishing.

## Data Availability

ZenodoExpanded Dataset for Two-Echelon Electric Vehicle Routing Problems (2E-EVRP) (Original data). Zenodo2E-EVRP-Instances (Original data).
